# Prognostic value of lymph node ratio in stage IIIC epithelial ovarian cancer with node-positive in a SEER population-based study

**DOI:** 10.18632/oncotarget.6911

**Published:** 2016-01-13

**Authors:** Juan Zhou, Zhen-Yu He, Feng-Yan Li, Jia-Yuan Sun, Huan-Xin Lin, San-Gang Wu, Qiong-Hua Chen

**Affiliations:** ^1^ Xiamen Cancer Center, Department of Obstetrics and Gynecology, The First Affiliated Hospital of Xiamen University, Xiamen, People's Republic of China; ^2^ Sun Yat-sen University Cancer Center, Department of Radiation Oncology, State Key Laboratory of Oncology in South China, Collaborative Innovation Center of Cancer Medicine, Guangzhou, People's Republic of China; ^3^ Xiamen Cancer Center, Department of Radiation Oncology, The First Affiliated Hospital of Xiamen University, Xiamen, People's Republic of China

**Keywords:** epithelial ovarian cancer, lymph node ratio, positive lymph nodes, prognosis, SEER

## Abstract

To assess the prognostic value of the lymph node ratio (LNR) in patients with stage IIIC epithelial ovarian cancer (EOC) with node-positive in a Surveillance, Epidemiology, and End Results (SEER) population-based study. Data of patients were obtained from the SEER database from 1990 to 2012, and analyzed using Kaplan-Meier survival methods and Cox regression proportional hazard model. The prognostic value of the LNR on cause-specific survival (CSS) and overall survival (OS) were calculated. A total of 5,926 patients were identified. Univariate analysis showed that the number of removed lymph nodes (RLNs), the number of positive lymph nodes, and the LNR were significantly associated with CSS and OS (*P* < 0.05 for all). Multivariate analysis indicated that a higher LNR was an independent prognostic factor for poorer CSS (hazard ratio [HR]: 1.896, 95% confidence interval [CI]: 1.709-2.104, *P* < 0.001) and OS (HR:1.679, 95% CI: 1.454-1.939, *P* < 0.001). Among patients with LNR ≤ 0.42 and those with LNR > 0.42, the 5-year CSS was 53.1% and 34.7%, respectively (*P* < 0.001), and the 5-year OS was 50.4% and 32.0%, respectively (*P* < 0.001). The prognostic value of the LNR persisted for patients after stratification by the numbers of RLNs, tumor histology, and tumor grade. LNR is a more accurate prognostic method for stage IIIC EOC patients. Patients with a higher LNR are associated with poorer survival in stage IIIC EOC.

## INTRODUCTION

Ovarian cancer has a high fatality rate and is the fifth leading cause of cancer mortality among women, with 21,980 new cases and 14,270 deaths in the United States during 2014 [1]. Most patients with ovarian cancer have advanced disease at the time of diagnosis because early-stage tumors are typically asymptomatic, resulting in a poorer long-time survival [2, 3]. Previous studies have shown that lymph node status can significantly affect the survival of patients with ovarian cancer [4, 5]. The International Federation of Gynecology and Obstetrics (FIGO) staging system considers lymph node status as an important factor for the staging of ovarian cancer. The FIGO categorizes patients with positive retroperitoneal lymph nodes as stage IIIC regardless of the intra-peritoneal extent of disease [6, 7]. Systematic lymphadenectomy was included in the FIGO guidelines because of the important prognostic value of lymph node metastasis. A meta-analysis indicated that lymphadenectomy can improve the survival of patients with advanced-stage epithelial ovarian cancer (EOC) [8]. However, the role of lymphadenectomy in EOC is still controversial [9-11]. The lymph node status of EOC patients is currently determined by the number of positive lymph nodes (PLNs), and this is affected by the number of resected lymph nodes (RLNs), and therefore may cause stage migration in some patients.

Lymph node ratio (LNR) is the ratio of the number of PLNs to the number of RLNs. Several studies have shown that a lower LNR is associated with a better prognosis in patients with breast cancer, esophageal cancer, gastric cancer, colon cancer, and pancreatic cancer [12-19]. However, data on the prognostic value of the LNR in EOC are still limited [20-22].

In the present study, we used a population-based Surveillance, Epidemiology, and End Results (SEER database to investigate the prognostic value the LNR in EOC, which may decrease the potential for selection and surveillance biases that are associated with single-institution studies.

## RESULTS

### Patient characteristics

Table 1 shows the demographic and clinicopathologic characteristics of the 5,926 patients with stage IIIC EOC who met the inclusion criteria. The median age was 59 years (range: 12 - 93 years), 87.0% (5153/5926) of patients were white, and 81.8% (4848/5926) had serous histology. The median number of RLNs was 10 (range: 1 - 88). The number of patients with 1-10, 11-20, and more than 20 RLNs was 2328 (39.3%), 2318 (39.1%) and 1280 (21.6%), respectively. Among all patients, the median number of PLNs was 2 (range: 1 - 69), and the median LNR was 0.38.

**Table 1:** Clinicopathological characteristics of patients with stage IIIC epithelial ovarian cancer with node-positive

Characteristic	n (%)
Age (years)	
Median (range)	59 (12-93)
≤50	1535(25.9)
> 50	4391 (74.1)
Race	
Black	317 (5.3)
White	5153 (87.0)
Others	456 (7.7)
Grade	
Well	274 (4.6)
Moderately	817 (13.8)
Poorerly	2938 (49.6)
Undifferentiated	1122 (18.9)
Unknown	775(13.1)
Histology	
Serous	4848 (81.8)
Mucinous	171(2.9)
Endometroid	512 (8.6)
Clear cell	343(5.8)
Undifferentiated	52(0.9)
Tumor location	
One site	2387 (40.3)
Paired site	142(2.4)
Bilateral site	3397 (57.3)
Number of RLNs	
Median (range)	10 (1-88)
Number of PLNs	
Median (range)	2 (1-69)
LNR	
Median (range)	0.38 (0.01-1)

### Univariate and multivariate analysis of prognosis

Univariate analysis (Table 2) showed that age, tumor grade, tumor histology, tumor location, RLN count, PLN count, and LNR were all significantly associated with both CSS and OS (*P* < 0.05 for all). Race had no significant effect on CSS and OS.

**Table 2:** Univariate cox regression analyses of patients with stage IIIC epithelial ovarian cancer with node-positive

Characteristic	CSS			OS		
	HR	95%CI	*P*	HR	95%CI	*P*
Age (years)						
≤50	1			1		
> 50	1.506	1.384-1.639	< 0.001	1.600	1.474-1.736	< 0.001
Race						
Black	1			1		
White	0.991	0.843-1.165	0.914	0.999	0.853-1.162	0.955
Others	0.881	0.717-1.083	0.229	0.880	0.722-1.072	0.203
Grade						
Well	1			1		
Moderately	2.215	1.751-2.802	< 0.001	2.126	1.709-2.645	< 0.001
Poorerly	2.654	2.128-3.310	< 0.001	2.519	2.052-3.091	< 0.001
Undifferentiated	2.688	2.130-3.391	< 0.001	2.522	2.031-3.132	< 0.001
Histology						
Serous	1			1		
Mucinous	1.072	0.874-1.316	0.502	1.113	0.920-1.346	0.271
Endometroid	0.732	0.641-0.835	< 0.001	0.760	0.671-0.861	< 0.001
Clear cell	1.295	1.117-1.503	0.001	1.351	1.175-1.553	< 0.001
Undifferentiated	0.750	0.502-1.121	0.161	0.758	0.519-1.108	0.152
Tumor location						
One site	1			1		
Paired site	1.687	1.337-2.128	< 0.001	1.594	1.275-1.993	< 0.001
Bilateral site	1.249	1.160-1.344	< 0.001	1.170	1.091-1.255	< 0.001
Number of RLNs (continuous variable)	0.992	0.989-0.994	< 0.001	0.991	0.988-0.993	< 0.001
Number of PLNs (continuous variable)	1.014	1.009-1.019	< 0.001	1.012	1.007-1.017	< 0.001
LNR (continuous variable)	2.006	1.823-2.208	< 0.001	1.967	1.795-2.155	< 0.001

Multivariate analysis (Table 3) showed that a higher LNR was significantly and independently associated with a poorer CSS (hazard ratio [HR]: 1.896, 95% confidence interval [CI] 1.709-2.104, *P* < 0.001) and OS (HR: 1.679, 95% CI: 1.454-1.939, *P* < 0.001). RLN and PLN count had no prognostic value in multivariate analysis. Multivariate analysis also indicated that age, tumor grade, histological type, and tumor location were significant prognostic factors.

**Table 3:** Multivariate cox regression analyses of patients with stage IIIC epithelial ovarian cancer with node-positive

Characteristic	CSS			OS		
	HR	95%CI	*P*	HR	95%CI	*P*
Age (years)						
≤50	1			1		
> 50	1.331	1.709-2.104	< 0.001	1.425	1.301-1.560	< 0.001
Grade						
Well	1			1		
Moderately	2.049	1.619-2.595	< 0.001	1.933	1.552-2.407	< 0.001
Poorerly	2.258	1.806-2.823	< 0.001	2.103	1.709-2.588	< 0.001
Undifferentiated	2.325	1.836-2.944	< 0.001	2.150	1.725-2.679	< 0.001
Histology						
Serous	1			1		
Mucinous	1.264	1.001-1.597	0.049	1.334	1.074-1.656	0.009
Endometroid	0.879	0.766-1.009	0.068	0.910	0.799-1.036	0.153
Clear cell	1.370	1.121-1.676	0.002	1.424	1.183-1.716	< 0.001
Undifferentiated	0.698	0.463-1.053	0.087	0.713	0.484-1.051	0.088
Tumor location						
One site	1			1		
Paired site	1.722	1.298-2.284	< 0.001	1.612	1.225-2.121	0.001
Bilateral site	1.257	1.159-1.364	< 0.001	1.199	1.110-1.296	< 0.001
Number of RLNs (continuous variable)	0.998	0.993-1.003	0.339	0.996	0.991-1.001	0.081
Number of PLNs (continuous variable)	1.007	0.998-1.016	0.144	1.008	0.999-1.017	0.070
LNR (continuous variable)	1.896	1.709-2.104	< 0.001	1.679	1.454-1.939	< 0.001

### Identification of optimal cut-off points of LNR

We used ROC analysis to determine the optimal cut-off point for prediction of CSS and OS based on the LNR. The results showed that 0.42 was the optimal cut-off point for CSS (Area Under roc Curve [AUC] = 0.603, *P <* 0.001) and OS (AUC = 0.609, *P <* 0.001). Therefore, a cutoff value of 0.42 was used as a prognostic factor for our subsequent analysis of the prognostic value of the LNR.

### Analysis of the prognostic impact of the LNR on survival

**Figure 1:**
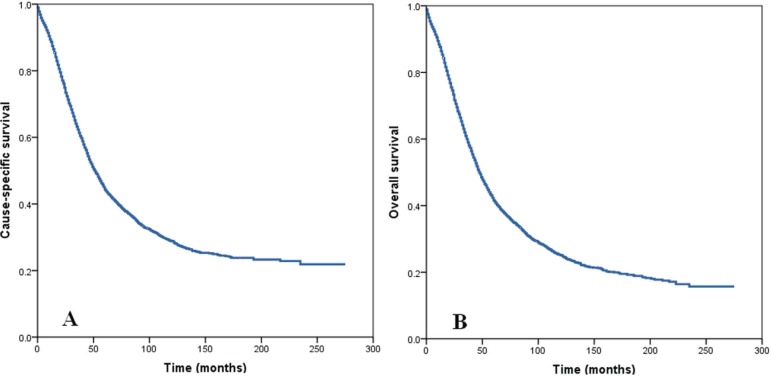
Cause specific survival **A.** and overall survival **B.** of patients with stage IIIC epithelial ovarian cancer with node-positive.

The median follow-up was 33 months (range: 1-275 months) in all patients. Among all patients, the 5-year and 10-year CSS was 44.6% and 29.0%, respectively (Figure 1A). The 5-year and 10-year OS was 41.8% and 25.3%, respectively (Figure 1B).

**Figure 2:**
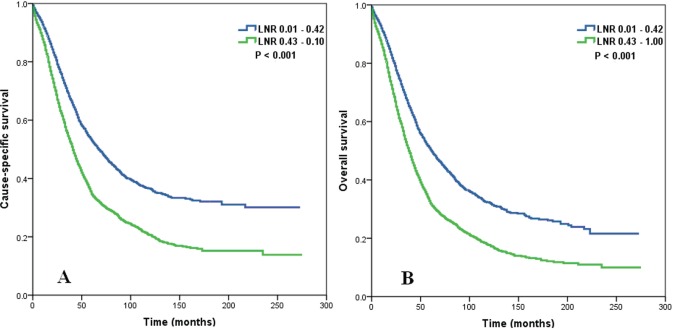
Impact of lymph node ratio on cause specific survival **A.** and overall survival **B.** of patients with stage IIIC epithelial ovarian cancer with node-positive.

The 5-year CSS was 53.1% for patients with an LNR ≤ 0.42 and 34.7% for those with an LNR > 0.42 (Figure 2A, log rank test: *P* < 0.001). The 5-year OS was 50.4% for patients with an LNR ≤ 0.42 and 32.0% for those with an LNR > 0.42 (Figure 2B, log rank test: *P* < 0.001).

We determined whether the influence of LNR on CSS and OS was modified by the number of RLNs. The results indicated that regardless of RLN count, a higher LNR was significantly associated with poorer CSS (log rank test: *P* < 0.001 for RLN count 1-10, *P* < 0.001 for RLN count 11-20, and *P* < 0.001 for RLN count >21) and OS (log rank test: *P* < 0.001 for RLN count 1-10, *P* < 0.001 for RLN count 11-20, and *P* < 0.001 for RLN count > 21).

We then examined the prognostic effect of the LNR according to tumor histology (serous vs. non-serous). In both serous and non-serous EOC, a higher LNR was significantly associated with poorer CSS (log rank test: *P* < 0.001 for serous histology, and *P* < 0.001 for non-serous histology) and OS (log rank test: *P* < 0.001 for serous histology, and *P* < 0.001 for non-serous histology).

We finally examined the influence of LNR on CSS and OS according to histologic grade. The results indicated that regardless of tumor grade, a higher LNR was significantly associated with poorer CSS (log rank test: *P* = 0.018 for well differentiated, *P* < 0.001 for moderately differentiated, *P* < 0.001 for poorly differentiated, and *P* < 0.001 for undifferentiated) and OS (log rank test: *P* = 0.018 for well differentiated, *P* < 0.001 for moderately differentiated, *P* < 0.001 for poorly differentiated, and *P* < 0.001 for undifferentiated).

## DISCUSSION

In the present study, we investigated the prognostic value of the RLN count, PLN count, and LNR in patients with stage IIIC EOC with node positive. Our results showed that a higher LNR was significantly and independently associated with poorer CSS and OS, and its prognostic value was superior to that of RLN count and PLN count.

Lymph node status is an important prognostic factor in patients with EOC. Currently, the FIGO staging system classifies EOC with PLNs as stage IIIC [6, 7]. Although it is easy to identify status of lymph nodes with negative or positive, but the number of PLNs may be affected by the total number of RLNs, and stage migration may occur if only a few lymph nodes are resected. Thus, incorrect staging would lead to improper treatment. Using LNR may reduce the potential bias due to variations of surgeons and pathologists which may affect accurate assessment of lymph node status.

There were three studies have assessed the prognostic value of the LNR in patients with EOC. Ataseven et al. investigated 809 patients with FIGO stage I-IV EOC and 398 patients with node positive. Their results showed that a higher LNR was independently associated with poorer OS (HR: 1.44, 95% CI: 1.04-2.00, *P* = 0.028), the 5-year OS rate was 42.5% for patients with an LNR of 0.25 or less, and 18.0% for patients with an LNR more than 0.25 (*P* < 0.001) [20]. Bachmann et al. investigated 95 patients with stage IIIC EOC, the results indicated a higher LNR was significantly association with poorer OS (*P* = 0.019), and the best OS was in patients with LNR of 0 to 0.5 [21]. Another study that used the SEER database examined 6,310 patients with stage IIIC-IV EOC and PLNs. The results showed that increasing LNR was significantly related to survival (*P* < 0.001), especially in patients with no macroscopic peritoneal disease [22]. The present study of stage IIIC EOC patients with node-positive also indicated that the LNR was an independent prognostic factor for survival.

The therapeutic value of systematic lymphadenectomy with advanced EOC is still unclear. Panici et al. performed the first multicenter randomized clinical trial, and the results showed that systematic lymphadenectomy was associated with significant improvement of progression-free survival, although OS was similar in the group that received systematic lymphadenectomy and the group that received resection of bulky nodes [5]. Based on these findings, it seemed that the total number of RLNs was not a reliable prognostic factor for patients with EOC. However, Pereira et al. used a mathematical model to predict the probability of a positive node in EOC surgical staging if at least 22 lymph nodes were removed between the pelvic and aortic lymphadenectomy [29]. Chan et al. used the SEER database to examine the impact of lymph node dissection on the survival of 13,918 women with stage III-IV node-positive EOC. They found that the higher number of RLNs was associated with a better survival, and the multivariate analysis indicated that the number of RLNs and the number of PLNs were significant and independent prognostic factors [30]. The German Association of Gynecological Oncology initiated the first study of advanced ovarian cancer (Lymphadenectomy in Ovarian Neoplasms) to compare the value of systematic lymphadenectomy with no lymph node resection in patients with no visible tumor residuals, in which systematic lymphadenectomy was defined as removal of at least 30 nodes (20 pelvic nodes and 10 para-aortic nodes [20]. In the present study, 39.3% of patients had 1-10 nodes, 39.1% had 11-20 nodes, and 21.6% had more than 20 nodes, and LNR had a significant prognostic value in each of these sub-groups. This indicates that the LNR may more accurately reflect lymph node status in EOC patients.

There is evidence that patients with EOC of different histological types and grades have different probabilities of lymph node metastasis [24-28]. Especially, patients with serous and poorly differentiated EOC have an increased risk for lymph node metastasis [24-28]. We used subgroup analysis to investigate the prognostic value of LNR in patients with EOC of different histological types and grades. The results showed that the LNR had prognostic value regardless of histological type and grade. Thus, in the lymph node staging of EOC, pN staging should not be confined to positive lymph nodes. Our findings indicate that the LNR has greater prognostic value than the RLN count and the PLN count. Thus, we suggest that the LNR should be considered in the lymph node staging of EOC.

There were several limitations in the present study. The main limitation of this study is the inherent bias that exists in any given retrospective study. Second, information about the volume of metastatic disease at diagnosis and therapeutic strategies (including the extent and outcome of primary surgery and the use and type of adjuvant chemotherapy) were not included in the SEER database. However, the strength of this study is that we analyzed the records of a large number of patients with node-positive EOC using the well-established SEER cancer registry, which is set up to reflect general population-based data [31]. In addition, few previous studies have investigated the role of the LNR in ovarian cancer, so there is no standard LNR cut-off point for comparisons of groups with lower and higher LNRs. In the present study, we used ROC analysis and determined 0.42 as the optimal LNR cut-off point. It is possible that other cut-off points are more applicable for other populations, and this must be confirmed by future studies with large sample sizes.

In conclusion, our results demonstrate that a higher LNR is significantly and independently associated with poorer survival in patients with stage IIIC EOC. LNR has a significant impact on survival in EOC patients than the number of RLN and PLN. Use of the LNR to characterize patients with EOC might be better predict outcomes, and compensate for deficiencies in the current staging system.

## PATIENTS AND METHODS

### Patients

Data were obtained from the SEER database, which consists of 18 population-based cancer registries. SEER data are an open access resource for cancer-based epidemiology and survival analyses. SEER*Stat software from the National Cancer Institute (SEER*Stat software, http://www.seer.cancer.gov/seerstat, Version 8.2.1) was used to identify eligible patients. Patients with diagnoses of EOC were identified from 1990 to 2012. We obtained permission to access these research data files with the reference number 11252-Nov2014 [23].

The following inclusion criteria were utilized for patient selection: *(i)* receipt of cancer-directed surgery (CDS) including lymphadenectomy, *(ii)* stage IIIC EOC with nodal positivity, *(iii)* pathological diagnosis of EOC with serous, mucinous, endometroid, clear cell, and undifferentiated histology. Pathologic diagnosis was based on the primary site using the International Classification of Disease for Oncology, Third Edition (ICD-O-3). Use of the SEER database does not require informed consent. This study was approved by the ethics committee of the First Affiliated Hospital of Xiamen University (Xiamen, China) and Sun Yat-sen University Cancer Center (Guangzhou, China).

### Clinicopathological factors

The covariates of demographic, clinicopathologic and treatment factors on the risk of cause-specific survival (CSS) and overall survival (OS) were extracted from SEER database.. These factors included age, race, histological type, histologic grade, tumor location, number of RLNs, number of PLNs, and the LNR. Vital status, including cause of death and follow-up duration were recorded.

### Statistical analysis

Univariate and multivariate Cox regression analyses were used identify significant risk factors for CSS and OS. Multivariable analyses examined factors that were significantly associated with CSS and OS in the univariate analyses. The optimal cut-off point for the LNR was determined from the receiver operating characteristic (ROC) curve. Calculation of survival rates were plotted by the Kaplan-Meier method, and compared using the log-rank test. All data were analyzed using the SPSS statistical software package, version 17.0 (IBM Corporation, Armonk, NY, USA). A *P*-value less than 0.05 was considered statistically significant.
